# Analyzing a putative enhancer of optic disc morphology

**DOI:** 10.1186/s12863-020-00873-z

**Published:** 2020-10-22

**Authors:** Vladimir Babenko, Roman Babenko, Yuri Orlov

**Affiliations:** 1 Institute of Cytology and Genetics, Lavrentyeva 10, Novosibirsk, 630090 Russia; 2 Novosibirsk State University, Pirogova Str 2, Novosibirsk, 630090 Russia; 3 I.M. Sechenov First Moscow State Medical University of the Ministry of Health of the Russian Federation (Sechenov University), Trubetskaya 8-2, Moscow, 119991 Russia

**Keywords:** GWAS, Enhancers, Optic disc size, CDC7, TGFB3, Glaucoma, 1000GP, GFI1, Alu, GTEx

## Abstract

**Background:**

Genome-wide association studies have identified the *CDC7-TGFBR3* intergenic region on chromosome 1 to be strongly associated with optic disc area size. The mechanism of its function remained unclear until new data on eQTL markers emerged from the Genotype-Tissue Expression project. The target region was found to contain a strong silencer of the distal (800 kb) Transcription Factor (TF) gene *GFI1* (Growth Factor Independent Transcription Repressor 1) specifically in neuroendocrine cells (pituitary gland). *GFI1* has also been reported to be involved in the development of sensory neurons and hematopoiesis. Therefore, *GFI1,* being a developmental gene, is likely to affect optic disc area size by altering the expression of the associated genes via long-range interactions.

**Results:**

Distribution of haplotypes in the putative enhancer region has been assessed using the data on four continental supergroups generated by the 1000 Genomes Project. The East Asian (EAS) populations were shown to manifest a highly homogenous unimodal haplotype distribution pattern within the region with the major haplotype occurring with the frequency of 0.9. Another European specific haplotype was observed with the frequency of 0.21. The major haplotype appears to be involved in silencing *GFI1*repressor gene expression, which might be the cause of increased optic disc area characteristic of the EAS populations.

The enhancer/eQTL region overlaps *AluJo* element, which implies that this particular regulatory element is primate-specific and confined to few tissues.

**Conclusion:**

Population specific distribution of *GFI1* enhancer alleles may predispose certain ethnic groups to glaucoma.

## Background

Genome-wide association studies (GWAS) identified thousands of common single nucleotide polymorphisms (SNPs) associated with complex diseases and quantitative traits [1]. These SNPs affect a trait in different ways. They cause an amino acid substitution, change the splicing process, and change the transcription rate or translational efficiency [2]. Identified variants were located in various regions of the genome including coding and regulatory genes regions. A large part of SNPs significantly associated with complex traits are located in non-coding regions: about 45 and 43% of such SNPs are located in introns or intergenic regions, respectively [1].

Gene regulation studies provide convincing evidence that a significant part of non-coding GWAS loci function as enhancers that can regulate specific genes up to 1 Mb away. The enhancers exhibit Transcription Factor Binding Sites (TFBS) clusters, open chromatin and corresponding histone marks. Thus, TFBS genome-wide locations identified by ChIP-Seq data, cell line specific chromatin state landscape, DNAse hypersensitive sites (DHS) at the intronic/intergenic sites can point to the enhancer loci. The results on the phenomenon were outlined in papers connected with the emergence of chromatin state segmentation routine [3–5]. As a practical outcome therein, the authors were able to associate a range of either target or tightly linked noncoding SNPs from GWAS studies with chromatin states corresponding to strong enhancers [4]. In particular, they identified two SNPs strengthening tissue – specific transcription factor binding sites: SNP rs9374080 associated with red blood cells (RBC) phenotype in close proximity (100 bp) to a strong enhancer in the K562 cell line, which reinforces the binding motif for *GFI1B*, a predicted repressor in K562, by strengthening it. Another lupus-associated SNP (rs9271055) locates within a lymphoblastoid (GM12878) strong enhancer and strengthens the binding motif for *ETS1*, a predicted activator of lymphoblastoid enhancers [4].

With an advent of Hi-C technology [6], Assay for Transposase Accessible Chromatin (ATAC-Seq) [7], and large-scale Multiplex Reporter Assays (MRA) [8], the enhancers identification was given renewed impetus resulting in the accumulation of a plethora of enhancer loci. VISTA, an experimentally verified enhancer source (https://enhancer.lbl.gov/) [9] includes around 2000 entries. Modern enhancer databases based on circumstantial evidence include entries on several million enhancers [10]. GeneHancer database appears to be the most comprehensive enhancer resource to-date [11]. It uses more than 1 million enhancers compiled from seven different genome-wide databases: the Encyclopedia of DNA Elements (ENCODE), Z-Lab Enhancer-like regions (http://zlab-annotations.umassmed.edu/enhancers/), the Functional Annotation of the Mammalian Genome (FANTOM) project [12], the Ensembl regulatory build [13], dbSUPER super-enhancers [14], EPDnew promoters [15] and UCNEbase of ultra-conserved non-coding elements [16].

The filtering criteria applied at the GeneHancer pipeline (as of 2018 version) underscores 285,000 enhancer loci, with 94,000 having more than one source of evidence (“double elite”).

The next crucial step in enhancer annotation (even more important than identification of enhancer itself) is annotating the genes associated with a particular enhancer. The Gene-GeneHancer associations were ascertained using 5 criteria/evidence sources:

1. eQTLs (expression quantitative trait loci) from The Genotype-Tissue Expression Consortium (GTEx; https://www.gtexportal.org/home/; [17]; version v6p)
2. Capture Hi-C promoter-enhancer long range interactions
3. FANTOM5 eRNA-gene expression correlations
4. Cross-tissue expression correlations between a transcription factor interacting with a GeneHancer and a candidate target gene;
5. Distance-based associations, including several approaches:
  - Nearest neighbors, where each GeneHancer is associated with its two proximal genes
  - Overlaps with the gene territory (intragenic)
  - Proximity to the gene TSS (< 2 kb)

In particular, eQTL database is a large-scale project of GTEx consortium [17]. It comprises 1,5 mln eQTL SNPs across 44 tissues (v.6p). Notably, GWAS SNPs often overlap the eQTL ones while not being themselves causative SNPs [18]. Thus, GWAS, chromatin state [19], and eQTL data complement each other with a task of elucidating causative SNPs in gene-enhancer interaction.

Speaking of gene-enhancer associations, there are currently numerous examples undermining the view, popular several years ago, that the majority of GWAS signals detected in intergenic/intronic regions are due to the functions of the nearest genes. For example, *FTO* intronic locus linked to obesity phenotype was proved to be a (800 kb) distal *IRX3* gene enhancer [20]. A range of intergenic enhancers is annotated in VISTA database [9].

This study is focused on optic disc area as a glaucoma-related trait. Previous research efforts have established 3 major traits impacting glaucoma risk rate: optic disc size (area), optic disc morphology, and retinal nerve fiber layer (RNFL) thickness [21].

At least four genome-wide association studies known to date (GWAS) [21–24] have demonstrated with high significance that rs1192415is associated with optic disc parameters (*P* < 8E-17; 3E-28; 8E-56; 6E-81, respectively).

In particular, SNPs rs1192415, rs4658101, and rs1192419 significantly associated with optic disc area have been localized between genes CDC7 and TGFBR3 [21–25]. The former encodes a cell division cycle protein with kinase activity that is critical for the G1/S transition and the latter encodes a transforming growth factor. The previous association of the above GWAS markers was majorly to the TGFBR3 gene [25]. This study will focus on the inferred function of the locus as *GFI1* gene enhancer.

The ultimate aim of the project was to analyze population specific distribution of genome-wide significant SNPs for optical disc area, which was not approached before.

## Results

### Choosing the region of interest

**Fig. 1:**
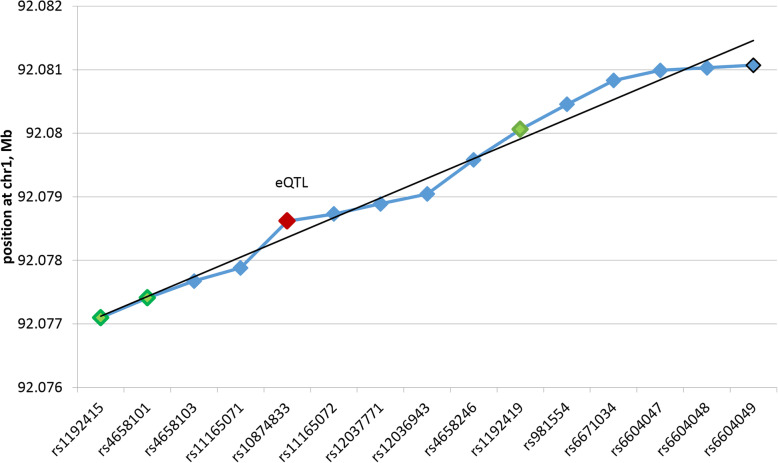
Chromosome location depicts evenly distributed 15 target SNPs within sequence span of 4 kb. Green shaded are GWAS SNPs; red shaded is the causal eQTL SNP (Table 1)

Several GWAS optic disc area projects identified ATOH7 gene and CDC7-TGFBR3 intergenic region as major determinants of optic disc area. According to the most recent meta-analysis studies the rate of associations is *P* < 1E-112 and *P* < 6E-81 for ATOH7 and CDC7-TGFBR3 intergenic region, respectively [24]. While ATOH7 gene was shown to be part of the embryonic optic disc area gene regulatory network, the locus association and mechanism of gene causality remained unclear. According to GWAS data, the locus represents a short 4 kb region starting with rs1192415 and ending with rs1192419 (Fig. 1).

The GTEx eQTL data repository emerged in 2015 [17] and proved to be the largest eQTL resource to date. It comprises 1.6 mln unique eQTL associations across 43 tissue types [17]. We queried GTEx data across our 4 kb region of interest (chr1 92.077mb – 92.082mb). It was found that the single tissue (pituitary gland) exhibits a set of SNPs associated with *GFI1*gene (eQTL *p*-value: 1.7 × 10^− 5^) located 840 kb downstream the target locus. *GFI1* gene is a growth factor gene largely involved in early embryogenesis of a subset of tissues, including retinal.

### GeneHancer elements associated with *GFI1*

From GeneHancer database nine enhancer elements manifesting *GFI1* as a target and located in the vicinity of *GFI1* (all GeneHancer elements are *cis* relative to a target gene(s)), GH01J091608 annotation corresponds to the target enhancer element. Single evidence of association of the latter with *GFI1* was ascertained via GTEx eQTL data as the only source.

Still, it maintains active state in 19 of 68 ENCODE cell lines/tissues (see ENSR00000009735 at www.ensembl.org), mostly related to embryonic stage (A549, Fetal Adrenal Gland, Fetal Stomach, H1-mesenchymal, H1-trophoblast, HMEC, HSMM, HSMMtube, HeLa-S3, IMR90, Left Ventricle, Lung, MSC (VB), NH-A, NHDF-AD, NHEK, NHLF, Osteoblasts, Placenta). The correspondent eQTL SNPs from GTEx exhibit a repressive effect for *GFI1* in only one of 43 tissues considered (pituitary gland). GeneHancer and Gene share a Topological Associated Domain (TAD) with evidence in 3/20 biosamples (HiC data).

### Distribution of alleles elucidated as eQTL locus

On the basis of SNPs data from both GWAS and GTEx we compiled a set of 15 SNPs (Table 1) evenly spanning within 4 kb (Fig. 1).

**Table 1:** 15 SNPs minor frequencies for merged GWAS and GTEx SNP sets considered in the study for European (EUR), South Asian (SAS), East Asian (EAS) and African (AFR) supergroups

	**EUR**	**SAS**	**EAS**	**AFR**	**maf allele**
rs1192415**	0.173	0.3037	0.1399	0.2103	G
rs4658101**	0.175	0.3037	0.1399	0.3829	A
rs4658103	0.3867	0.3507	0.1399	0.4276	G
rs11165071	0.3867	0.3517	0.1399	0.4276	G
**rs10874833**^a^	0.3877	0.3517	0.1399	0.4375	C
rs11165072	0.3877	0.3517	0.1399	0.4375	T
rs12037771	0.3946	0.3569	0.1399	0.4375	T
rs12036943	0.3877	0.3517	0.1399	0.4375	A
rs4658246	0.3877	0.3517	0.1399	0.4375	T
rs1192419**	0.1759	0.3047	0.1339	0.2927	A
rs981554	0.3857	0.3282	0.1359	0.4355	A
rs6671034	0.3867	0.3272	0.1339	0.4365	A
rs6604047	0.3867	0.3262	0.1339	0.4365	A
rs6604048	0.3867	0.3272	0.1339	0.4365	T
rs6604049	0.3867	0.3272	0.1339	0.4365	A

**GWAs SNPs with minimal *P* values: (*P* < 8E-17; 6E-81; 8E-56), respectively

^a^GTEx causal SNP

**Fig. 2:**
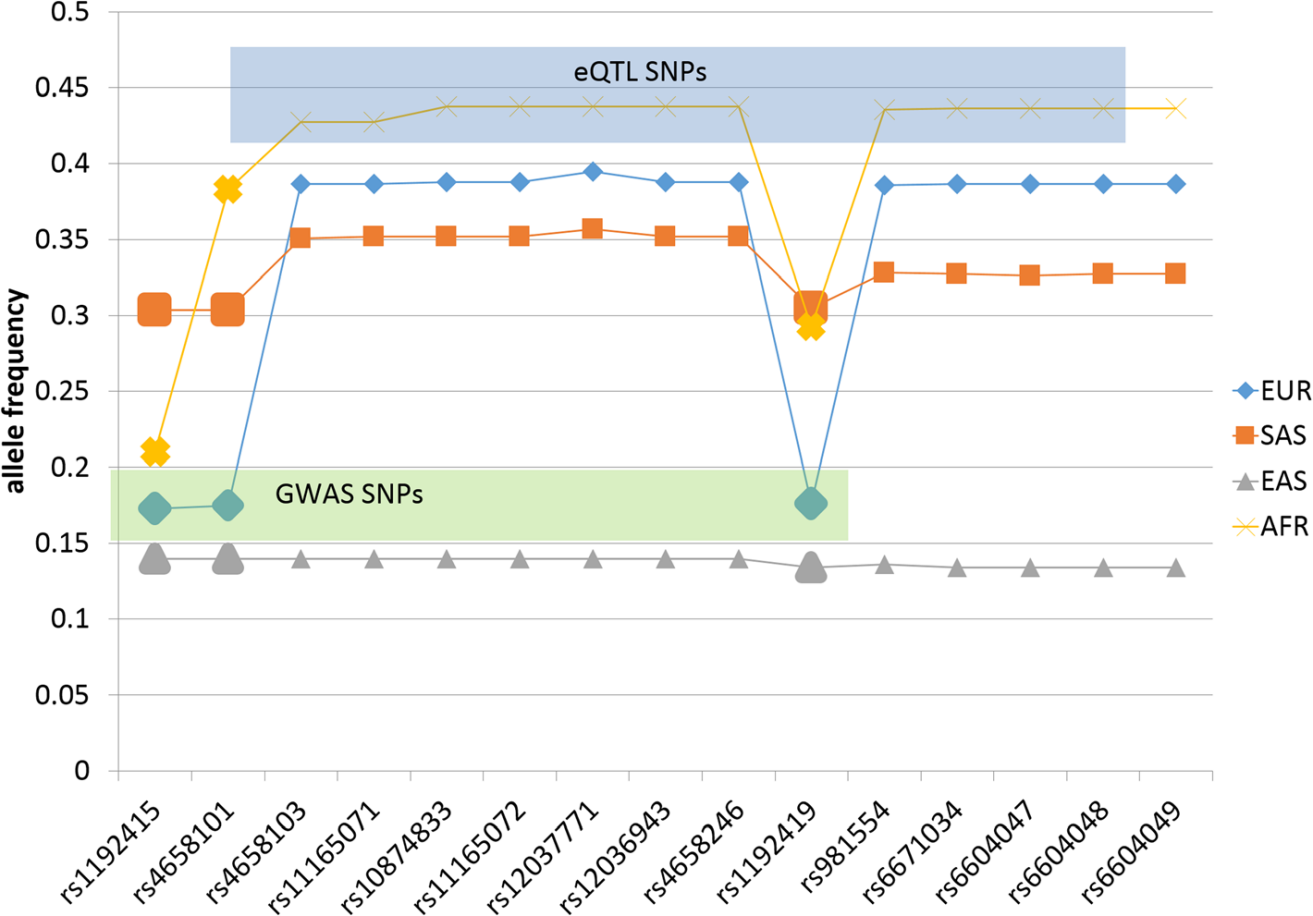
Graphic representation of MAF frequencies from Table 1 for European (EUR), South Asian (SAS), East Asian (EAS) and African (AFR) supergroups. GWAS SNPs are plotted with enlarged markers

The profiles of MAF alleles across all supergroups (Fig. 2) underscore that GWAS related haplotype outstands from haploblock linkage in all supergroups other than EAS. Overall, the region maintains a high linkage rate (*r* > 0.9) for all eQTL SNPs, as well as within GWAS SNPs(*r* > 0.9).

The highly interlinked 4 kb locus has proved to be eQTL allele (Fig. 2; blue shaded) for the *GFI1* gene. Three GWAS SNPs differed in their MAF values from the core haplotype in EUR populations (Fig. 2; enlarged blue markers, green shadowed), and thus are non-equally linked with others (*r* < 0.8) in the supergroups other than EAS (full linkage overall), while highly interlinked with each other (*r* > 0.9) in European and South Asian supergroups. SNPs are sorted according to their order on the chromosome.

### Distribution of haplotypes

We retrieved 15-fold haplotypes frequencies from 1000G phased data for each of the supergroups (Table 2) that are higher than 0.1 total across all populations. Due to a high linkage rate within the locus, three haplotypes encompass > 95% of variance in all supergroups except AFR (Table 2). All eQTL SNPs are completely linked and represent 2 dichotomous alleles. The major haplotype exhibits no variation and is interlinked with GWAS SNPs, so all variations reside in minor allele spectra. The haplotypes composition manifests overlapping GWAS 3-fold haplotype with completely linked 12-fold eQTL haplotype. Thus, as a major (tag) haplotype we may set the highest associated with trait GWAS polymorphism rs4658101 (Table 1) complemented with most ‘enhancer’ manifested, presumably casual, eQTL SNP rs10874833.

**Table 2:** Distribution of top haplotypes frequencies in 4 major supergroups^a^

**Haplotype (15 letters)**	**‘Tag’ haplotype**	**AFR**	**EAS**	**EUR**	**SAS**
**AG**AA***G***GCGA**G**GGGAG	GG	0.562	0.86	0.605	0.642
**GA**GG***C***TTAT**A**AAATA	AC	0.209	0.132	0.172	0.28
**AG**GG***C***TTAT**G**AAATA	GC	0.043	0	0.21	0.045
SUM:		0.814	0.992	0.987	0.967

^a^ Supergroup denotations are given in Fig. 2. GWAS SNPs are marked with bold, eQTL causal SNP is marked in bold Cyrillic. The first allele is a major one (*f > 0.5* in all supergroups), two others are minor ones. The ‘tag’ haplotype represents convoluted GWAS/eQTL 2-letter haplotype of tag SNPs (pos. 5:rs4658101, pos. 10:rs10874833). Two African–specific low frequency haplotypes **AA**GG***C***TTAT**G**AAATA (0.099) and **AA**GG***C***TTAT**A**AAATA (0.07) were omitted from consideration according to selection criterion

**Fig. 3:**
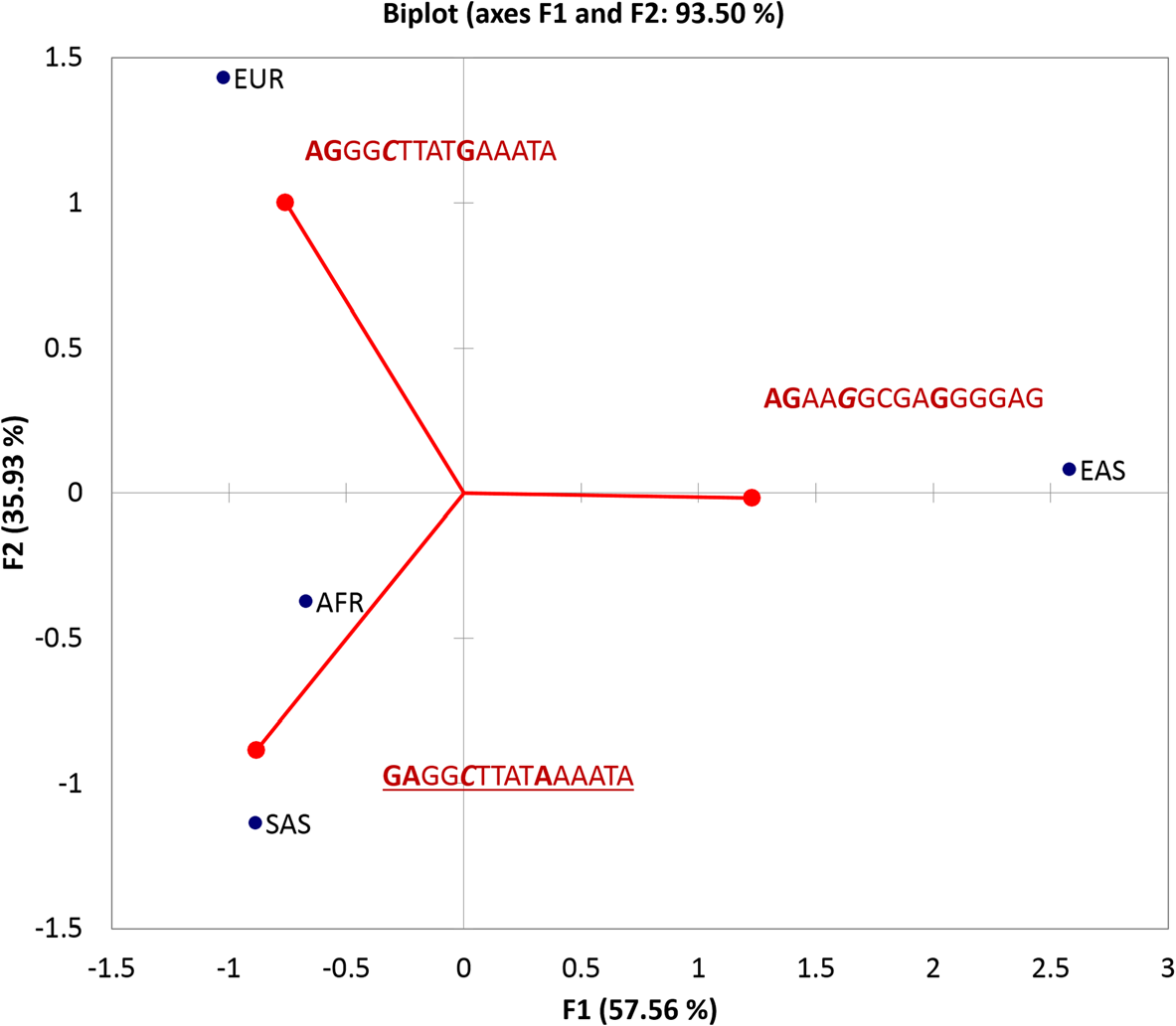
PCA plot of 3 major haplotypes (Table 2) in European (EUR), South Asian (SAS), East Asian (EAS) and African (AFR) supergroups. GWAS SNPs (Table 1) are marked in bold. eQTL casual repressive SNP rs1087483 G- > C (pos. 5) is marked in bold italics. Underlined is the high-risk allele according to GWAS reports. The low-risk allele (GWAS haplotype: **AGG**; on the right) is highly elevated at EAS supergroup, and is present at least at 50% in the others (Table 2)

After performing PCA for Table 2 data, we found the haplotype spectra quite distinctly distributed across 4 major continental supergroups (Fig. 3), implying it can affect specifically the Asian populations due to a single mode haplotype distribution with highly linked optical disc size allele and eQTL SNPs. Also, we may underscore the unique European–descent haplotype, which apparently disrupts the silencer site (eQTL) (Fig. 3).

### Allele dosage estimation

If the causal eQTL SNP rs1087483 G is considered as the risk one (it doesn’t decrease *GFI1* expression) and co-dominant, then the estimated homozygous state in EAS would be p (GG) = 0.74 within the major haplotype. In contrast, in EUR supergroup it would be p (GG) = 0.36, 2 times lower than in EAS. Overall, the major allele in EUR, AFR, SAS supergroups occurs with approximately similar frequency (Table 2).

## Discussion

The advance of the resources and technologies on enhancer validation made it possible to move the task of annotating GWAS associated loci toward a new stage. While the information on the enhancers, and especially, their target regions is still far from complete, the range of targets conceived to date have been ascertained.

We studied the CDC7-TGFB3 intergenic region to elucidate the exact target of a 4 kb loci enriched with GWAS SNPs associated with optical disc size, at the same time manifesting eQTL SNPs pointing to *GFI1* gene target at least in pituitary gland tissue based on eQTL GTEX database evidence. We justify the likelihood of this enhancer to be also active in embryonic retinal tissues by presenting two observations.

**Fig. 4:**
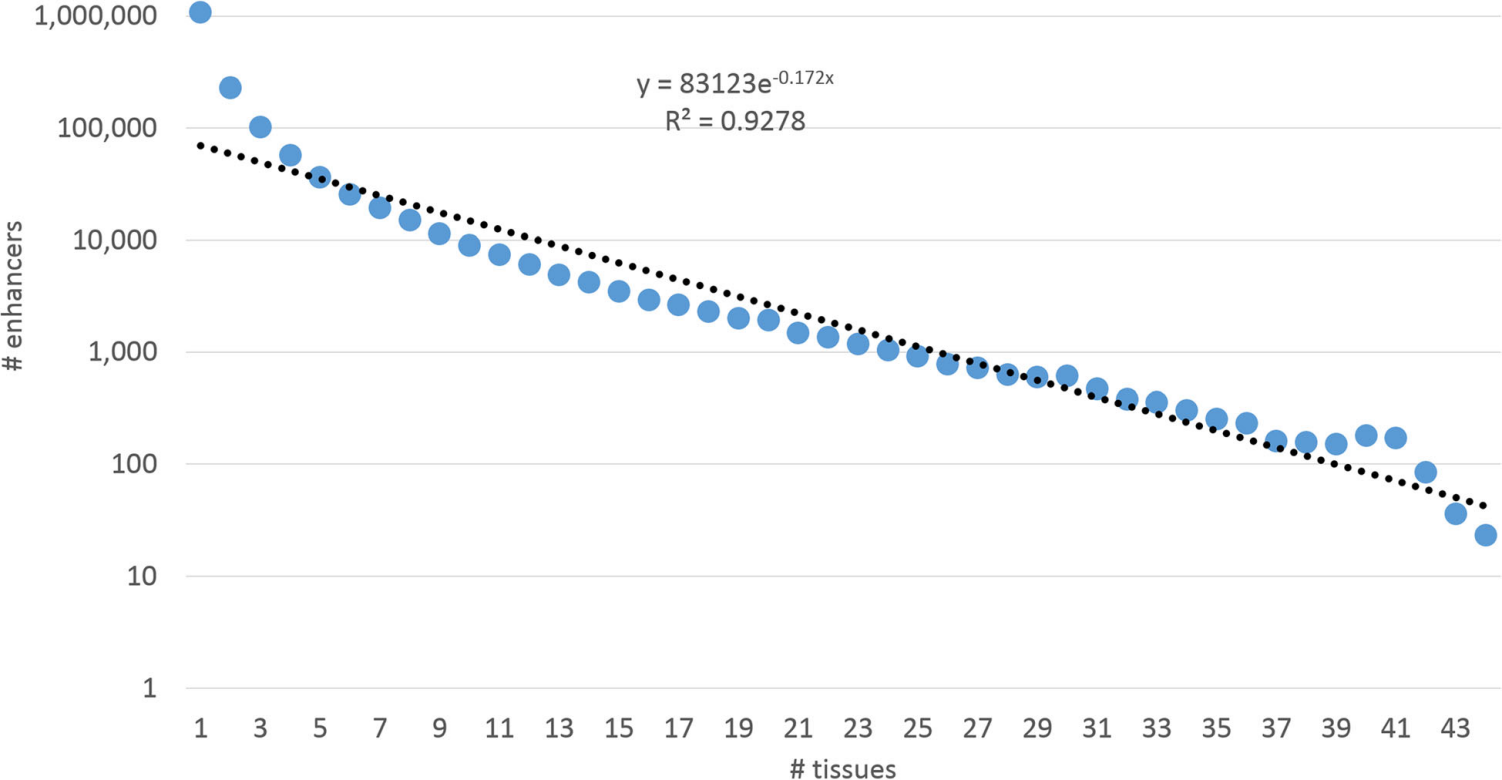
Distribution of enhancers’ number given the number of tissues it is active in, and its exponential approximation based on GeneHancer elements database

First, we assessed the tissue specific distribution of eQTL regions against the number of tissues they are active. As we can see from Fig. 4, there is a strong tenfold overrepresentation of single-tissue eQTL regions not fitting exponential distribution, otherwise highly concordant (*R*^*2*^ = 0.93, *df* = 42; *p* < 2.6E-51).

We assume that overabundance of single – tissue enhancers implies they may get activated at certain embryonic stages, or in some specific tissues not currently presented in GTEX tissue panel.

While there is a promising approach for detecting embryonic enhancers reported quite recently [26], it may require some time to address the issue explicitly.

As the second observation corroborating the hypothesis stated above, we correspond that none of the distinct enhancers reported in GeneHancer database overlap each other. That means that virtually no random overlapping may occur for the embryonic-related CDC7-TGDBR3 enhancer and pituitary specific one.

Based on these observations, we speculate that the pituitary specific enhancer may possibly get activated in embryonic nerve tissue in the course of optic disc development.

*GFI1* was first ascertained as an oncogene in lymphomas [27, 28]. Further studies showed that *GFI1* mRNA is expressed in many precursors that give rise to neuronal cells during embryonic development in mouse [29]. *GFI1* mRNA is expressed both in the CNS and PNS, featuring many sensory epithelia cells including the developing retina, the eye, presumptive Merkel cells, the lung and hair cells of the inner ear [30]. It has homologues in fly (*senseless*) and worm (*pag-3*).

*GFI1* was proved to maintain tissue specific distal enhancers up to 100 kb in early hematopoietic cells [31], later ascertained to be involved in a complex interplay of enhancers and silencers, as well as genes within Gene Regulation Networks in hematopoietic lineage specification [32]. Similarly, *GFI1* plays a crucial role during embryogenesis of other tissues [33]. During embryogenesis the enhancers’ network is one of the expanded regulatory networks and often incorporates Transposable Elements (TE) including *Alus*, which later become repressed [34–36]. Currently, GeneHancer resource provides evidence (eQTL only) for the target region as an enhancer of *GFI1* [11].

**Fig. 5:**
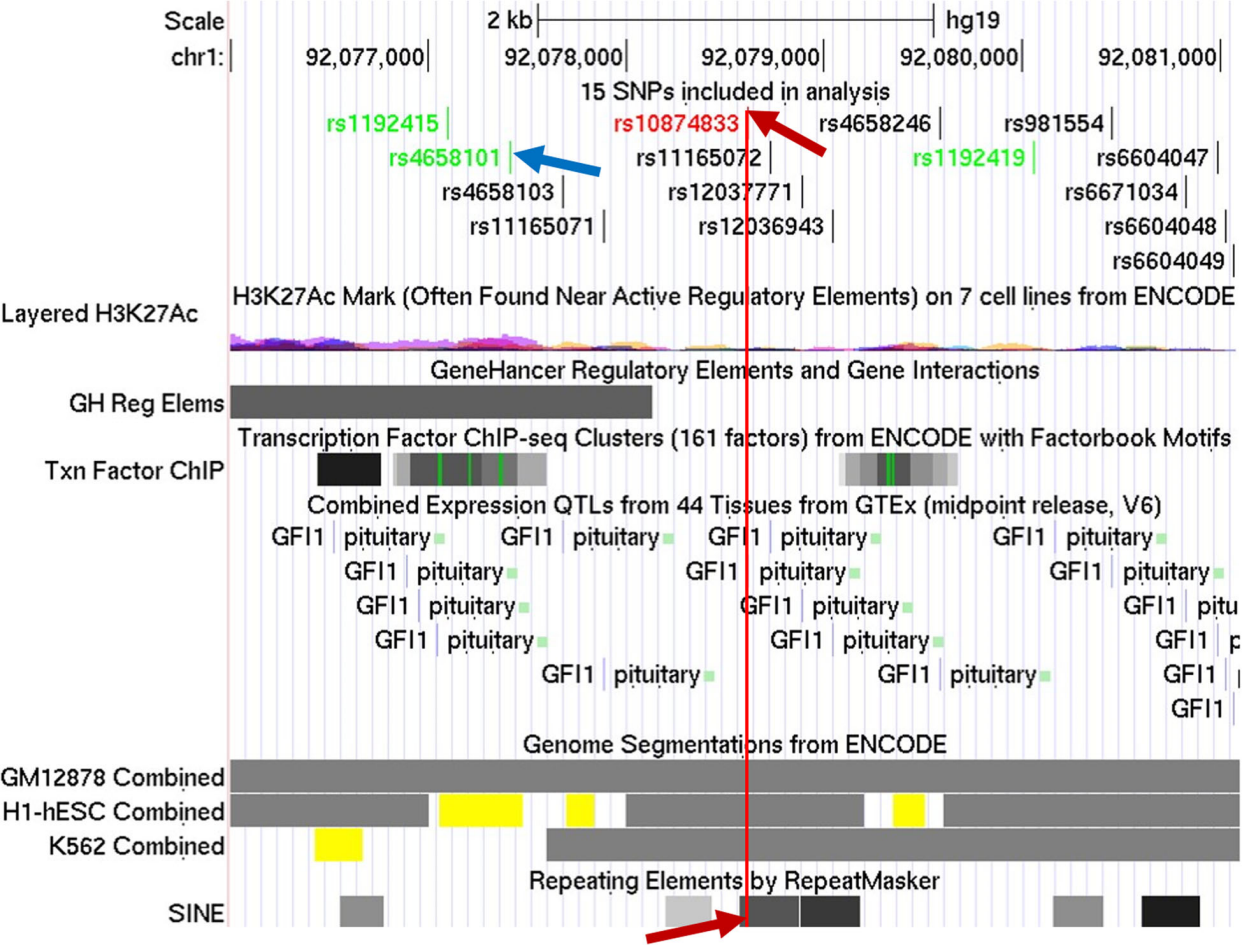
Fifteen target SNPs (top track) spanning the region enriched with enhancer histone marks (yellow boxes manifested in H1-hESC cell line) comprising 3 GWAS SNPs (green shaded in the top track) and 12 GTEx eQTL SNPs (top track; “GFI1 pituitary” labels, middle track), as well as 6Alu sequences (bottom track). Marked are highest ranked SNPs (rs4658101, blue arrow, and rs10874833, red arrow; another one pointing to target *Alu*5’ region, bottom track). Enhancer GH01J091608 (‘GeneHancer Regulatory Elements’ track, middle positioned; grey shaded box) overlaps the yellow shaded box

The tissue restricted enhancer in the region spanning target GWAS SNPs (Fig. 5; yellow track for hESC cell line) comprises several TFBS, including *CTCF* and *p300,* which regularly accompany Hi-C looping factors [11]. It looks probable that the enhancer site changes chromatin context in the locus vicinity upon activation and accomplishes interaction of the locus with the promoter region of *GFI1* via Hi-C mechanism. Since GWAS SNPs are also heavily interlinked with eQTL SNPs which decrease the expression of *GFI1* (Table 1), we speculate that three *GFI1* expression alteration hypotheses are possible: 1) GWAS SNPs are the cause of abrogation of the aforementioned looping, e.g. by disruption of CTCF or other binding sites, resulting in the alteration of *GFI1* expression; 2) *GFI1* binding site located in *AluJo/AluSo* segment [37] is probably involved in local autoregulation of *GFI1* expression. Note that the causative eQTL SNP rs10874833 is only 10 bp away from the core consensus of *GFI1* within *AluJo* (‘AATC’; [37]) and may affect the binding affinity of *GFI1* to this site; 3) *AluJo* comprises CTCF binding site which is altered by eQTL SNP followed by long-range interaction abrogation (loop disruption).

Since zinc finger protein GFI1 contains at least 3 zinc finger motifs, the consensus should be much more extended, and it is partially reflected in overall consensus length of 10 positions in Hocomoco database (Additional File 1), though other positions are not informative probably due to (single dependence) weight matrix approach utilized in Hocomoco [38], not accounting for possible interaction of the positions, including palindromic structure preference [39], and, more importantly for the ZNF-related TFs, the orientation-related specifics mediated by specific pairwise position correlations [40]. It should be noted the *Alu* related abundance of *GFI1* motif.

The target locus maintains specific structure comprising tandem *Alu* pair flanked with enhancer- specific H3K27ac histone marks (Fig. 5). At least 4 eQTL SNPs fall into 3 *Alus,* including the causal eQTL SNP (rs1087483; Fig. 5, red arrow). Since eQTL SNPs are highly interlinked in the region in all supergroups, we may speculate that since the highest ranked SNPs, both GWAS and eQTL ones (rs4658101 and rs10874833, respectively; Fig. 5) belong to the region flanked with enhancer border marks (Fig. 5, yellow boxes of chromatin segmentation track), other SNPs outside the region are presumably non-causative.

As mentioned above, we should add that GFI1 TFBS residing in a transposable element like *Alu* is an annotated event [29, 38], and suggests multiple factors, including DNA methylation and heterochromatization that may play a role in accessibility of such a site in a tissue and stage-specific manner. It is worth noting that *Alu* TEs proved to be involved in developmental stages [37], and comprise the following TFBSs: GFI1 (V$GFI1.01), PITX2 (PITX2.Q2), PAX6 (PAX6.01), SIX3 (SIX3.01) for the factors implicated in eye development [38], as well as in Hi-C conformation [41, 42]. Notably, eQTL causative SNP rs10874833, being located within the GFI1 TFBS in *Alu*, may disrupt the binding site of GFI1, which can be the cause of negative feedback on *GFI1* expression rate reported in GTEx survey.

Using GTEX v8 RNA-Seq profiles we also ascertained that *GFI1* highest expression rate across 53 tissues available is maintained in EBV-transformed lymphocytes (cell culture): its average expression rate is 2.16 Tpm while standard deviation being 0.64 (100 samples considered). Notably, *GFI1* gene maintains high expression correlation rate with another oncogene *Malat1* ncRNA, while both maintain negative correlation to *TP53* (Table 3) as reported earlier [43]. It most probably reflects EBV induced immortality in cells. *GFI1* expression rate in pituitary tissue is about 0.13 (±0.08) Tpm. While this value is more than a median across 53 tissues, still it’s rather low for the robust assessments of its interactions/pathways, implying a necessity of addressing embryonic cells for the final verdict.

**Table 3:** Pairwise Pearson correlation rate of three genes based on 100 random samples of EBV-transformed lymphocytes RNA-Seq data (GTEx Consortium; v.8; https://www.gtexportal.org)

Variables	GFI1	TP53RK	TP53	MALAT1
GFI1	**1**	−0.222	−0.058	**0.386**
TP53RK	−0.222	**1**	**0.410**	**−0.405**
TP53	−0.058	**0.410**	**1**	**−0.331**
MALAT1	**0.386**	**−0.405**	**−0.331**	**1**

Values in bold are different from 0 with a significance level alpha = 0.05

It’s worth noting that pituitary gland contains stem/progenitor cells pool [44], and anatomically is located within close vicinity of the optical chiasm [45], which may imply common mechanisms of their early embryonic development, including retinal tissues. Pituitary tissue is the top one in *Malat1* expression rate throughout the 53 tissues maintained by GTEX Consortium v.8 dataset (personal observation), underlining its high stem cell specific genes turnover [43].

Still, it is necessary to state that the target enhancer score in current GeneHancer database version is small (score = 232 from 700 maximal) accounting for the distance to the target gene (840 kb), *GFI1* small expression rate in the tissues considered (see above), sparse tissue manifestation instances and single (eQTL) source of evidence.

Along with gaining the insight into the target and mechanisms for GWAS intergenic locus supported by GTEx eQTL data considering optic disc size trait, we also performed haplotyping of GWAS encompassed locus for 4 supergroups. It was elucidated that EAS supergroup maintains the unimodal allele frequency distribution spanning 15 SNPs belonging to the major low-risk allele. But it also implies ‘G’ eQTL allele at causative SNP, and thus probably maximizing the optic disc size trait leading to the elevated myopia incidence observed in EAS populations [24]. Note, that on the contrary, AFR, SAS supergroups maintain elevated frequency of risk associated allele (Table 2; Fig. 3). Also, AFR supergroup maintains African- specific low frequency haplotypes with GWAS SNPs triplets AA(G/A) and causal eQTL SNPs to be a ‘C’ variant (Table 2, Caption), which possibly impacts the elevated rate of glaucoma incidence in this group as well.

## Conclusion

Besides outlining that *Alu* TEs are apparently involved in regulation of the target site, we report unequal frequency profiles of GWAS/eQTL enhancer-related haplotypes in continental supergroups (Table 2, Fig. 3), implying the optical disc area trait may pertain to the specific correspondent enhancer site involvement. We underscore the presence of unique European specific minor haplotype along with high homogeneity for major haplotype in East Asian population. Based on the observation, we speculate that there may be 2 alternative factors modulating enhancer effects for the eye disc area. To assess it, it would be reasonable to experimentally elaborate on haplotypes consisted of 2 tag SNPs (Table 2). It may shed the light if there are specific risks of each haplotype state.

Concerning the possible implications in other GWAS/eQTL functional annotations, we’d underline the essential obstacles of inherently developmentally onset traits analysis rendering assessment of gene expression rate dynamics at specific tissues/organs developmental stages. Extensive chromatin remodeling dynamics during embryogenesis also adds to the complexity of developmentally onset traits analysis.

## Methods

### eQTL data

GTEx data of eQTL score profiles [17] in the region were downloaded from UCSC resource (www.genome.ucsc.edu; genome.ucsc.edu > exeqtlcluster)

GeneHancer data on the promoter and enhancer regions were downloaded from ucsc resource (www.genome.ucsc.edu; genehancer_reg_elements_doubleelite track)

### 1000 Genomes data

We downloaded a subset of 1000 Genomes Consortium (1000GP) phase 3 data [46] (http://www.internationalgenome.org/) for 4 supergroups: 1) African (AFR; 504 individuals total); 2) East Asian (EAS; 504 individuals total), 3) European (EUR; 503 individuals total), 4) South Asian (SAS; 489 individuals total). We omitted American native supergroup (AMR) from 1000G since it significantly overlaps with other 4 supergroups by alleles profiles ([46], personal observation). A total of 2000 individuals were analyzed. The data are presented in Additional File 2.

### Retrieval and statistical tools

We employed the PLINK toolset [47] for extracting and managing the haplotype data. We used XLStat software for Principal Component Analysis (PCA; www.xlstat.com).

## Supplementary information

**Additional file 1. **Segment of *AluJo* is the preferred GFI1 binding site. Supplementary information.

**Additional file 2.** Data on 1000G 15-fold SNPs haplotypes of 2000 individuals studied.

## Data Availability

All data generated or analyzed in this study are publicly available: 1000 Genomes Consortium at ftp://ftp-trace.ncbi.nih.gov/1000genomes/ftp; and UCSC resource at: http://hgdownload.soe.ucsc.edu/goldenPath/hg19/database/. Additionally, the pre-processed data for 15 SNPs and 2000 individuals from 1000GP is located in Supplementary File 2.xls.

## Abbreviations

TE: Transposable Elements
TFBS: Transcription Factor Binding Sites
*GFI1*: Growth Factor Independent 1
