# Correction to: Analyzing a putative enhancer of optic disc morphology

**DOI:** 10.1186/s12863-020-00929-0

**Published:** 2021-02-04

**Authors:** Vladimir N. Babenko, Roman O. Babenko, Yuriy N. Orlov

**Affiliations:** 1 Institute of Cytology and Genetics, Lavrentyeva 10, Novosibirsk, 630090 Russia; 2 Novosibirsk State University, Pirogova Str 2, Novosibirsk, 630090 Russia; 3 I.M. Sechenov First Moscow State Medical University of the Ministry of Health of the Russian Federation (Sechenov University), Trubetskaya 8–2, Moscow, 119991 Russia

**Correction to: BMC Genet 21, 73 (2020)**

**https://doi.org/10.1186/s12863-020-00873-z**

Following publication of the original article [1], the authors reported that the author names have been spelled incorrectly in the published article.

The corrected names can be found in this correction article.

The authors apologize for any inconvenience caused.
