# Supplementary material for: Analyzing a putative enhancer of optic disc morphology
Source: BMC Genet. 2020 Oct 22;21(Suppl 1):73. doi: 10.1186/s12863-020-00873-z (PMC7583307; doi:10.1186/s12863-020-00873-z)
Supplement: Supplementary file 1 — Additional file 1. Segment of AluJo is the preferred GFI1 binding site. Supplementary information. [file 12863_2020_873_MOESM1_ESM.pdf]

Segment of AluJo is the preferred GFI1 binding site. Supplementary information

As long as in 2003 it was established that *GFI1* maintains strong affinity to the sequence: GCTCAATG**CCTGTAATCCCAGCACTTTGGGA**A*G*GCAGGCG (Duan, Horwits, 2003; italic shaded 'G' corresponds to casual eQTL SNP: rs10874833). It was deposited in Institute for Transcriptional Information Transcription Factor database at *IFTI-Mirage website* (www.ifti.org; site id: S07858, S07853 (complement)). The site was later identified as a segment of *Alu* sequence, which indeed is a regular target site of *GFI1* (Polak, Domany, 2006). It contains a core motif (AATC, yellow-shaded above), which was later on used as a basic consensus motif for *GFI*1 (GFI1_HUMAN.H11MO.0.C; Hocomoco database; Kulakovskiy et al., 2018; Fig. 1).

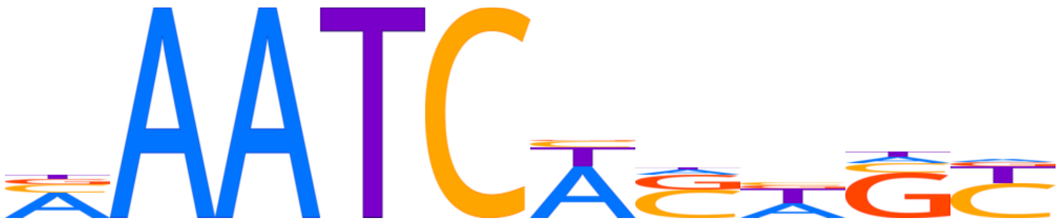

Fig. 1. Logo GFI1 binding consensus corresponding to Hocomoco entry (Kulakovski et al., 2018).

We performed the 40bp motif search against human genome sequence by blat tool (Kent et al., 2006) and ascertained that exact matches were found in only 30 instances in human genome, including the unique instance (chr1: 92078573-92078616; hg19) within the target region.

More extended consensus has been reported in TRANSFAC Database (Wingender, 2008) V$GFI1.01: AATC..AG. We aimed at assessing the major position of the core consensus V$GFI1.01 in *AluJo*, *AloJb* sequences. By running a pattern match against *AluJo* sequences we found that they are: pos 65 (*AloJo*) and pos 35 (*AluJb*) (Fig. 2),

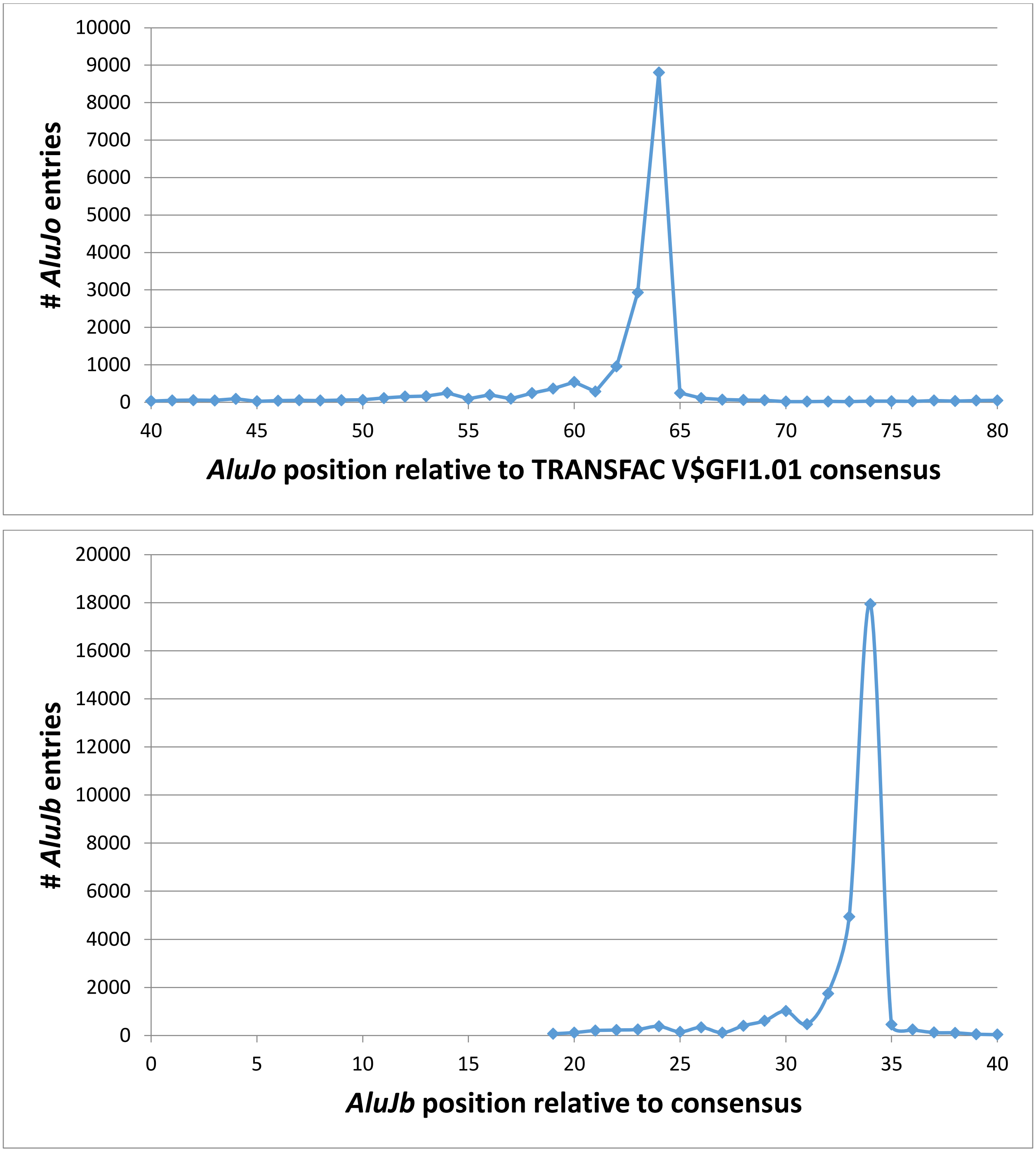

Figure 2. Identification of TRANSFAC V$GFI1.01 consensus (AATC..AG) positions in AluJo (A) and AluJb (B). Phased (pos 65) of the *GFI1* AATC..AG TFBS consensus underlines principal points in AluJo sequences found essentially at the pos 65 (9000 from 22000 found by pattern ‘AATC..AG’, TRANSFAC database binding motif name: **V$GFI1.01**; total scanned: 68000; presence ratio 0.32 in direct strand (**0.64** in both)); AluJb sequences maintain it at pos 35

(18000 from 37000 found by pattern: 'AATC..AG'; total scanned: 134,000; ratio: 0.28 in direct strand (**0.56** in both)).

We built logo consensus for the phased *AluJo* sequences presented in Figure 3.

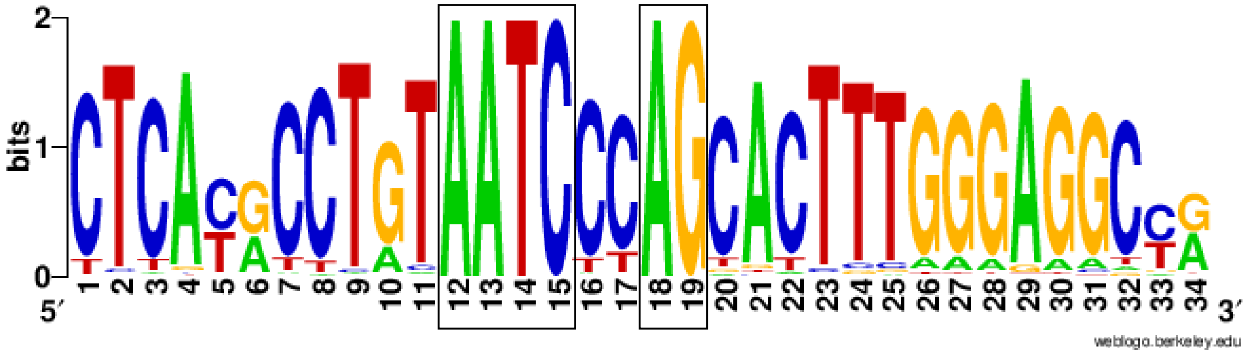

Fig. 3. Expanded consensus of TRANSFAC V$GFI1 (squared out) in *AluJo* phased sequences. Position 31 at the logo corresponds to casual eQTL SNP: rs10874833 (G->C)

Conslusion: We observe a cg-rich sequence typical for ZNF-binding sequence and it is quite plausible that G->C polymorphism at pos 31 close to the core may alter the binding affinity of GFI1 to the sequence.

References

1. Duan Z, Horwitz M. Gfi-1 oncoproteins in hematopoiesis. Hematology. 2003 Oct;8(5):339-44. Review. PubMed PMID: 14530176.
2. Polak P, Domany E. Alu elements contain many binding sites for transcription factors and may play a role in regulation of developmental processes. BMC Genomics. 2006; 7:133.
3. Wingender E. The TRANSFAC project as an example of framework technology that supports the analysis of genomic regulation. Brief Bioinform. 2008; 9(4):326-332. doi: 10.1093/bib/bbn016.
4. Kulakovskiy IV, Vorontsov IE, Yevshin IS, Sharipov RN, Fedorova AD, Rumynskiy EI, Medvedeva YA, Magana-Mora A, Bajic VB, Papatsenko DA, Kolpakov FA, Makeev VJ. HOCOMOCO: towards a complete collection of transcription factor binding models for human and mouse via large-scale ChIP-Seq analysis. Nucleic Acids Res. 2018 Jan 4;46(D1):D252-D259. doi: 10.1093/nar/gkx1106.
